# Deproteinization of Chitin Extracted with the Help of Ionic Liquids

**DOI:** 10.3390/molecules27133983

**Published:** 2022-06-21

**Authors:** Douglas R. Lyon, Bryan R. Smith, Noureddine Abidi, Julia L. Shamshina

**Affiliations:** 1 Lummus Technology, Houston, TX 77086, USA; dlyonjr1@gmail.com; 2 InBio, Charlottesville, VA 22903, USA; bsmith@inbio.com; 3 Fiber and Biopolymer Research Institute, Texas Tech University, Lubbock, TX 79409, USA

**Keywords:** ionic liquid, chitin, deproteinization, Polysorbate 80 (Tween^®^ 80)

## Abstract

The isolation of chitin utilizing ionic liquid 1-ethyl-3-methylimidazolium acetate has been determined to result in polymer contaminated with proteins. For the first time, the proteins in chitin extracted with ionic liquid have been quantified; the protein content was found to vary from 1.3 to 1.9% of the total weight. These proteins were identified and include allergenic proteins such as tropomyosin. In order to avoid ‘traditional’ hydroxide-based deproteinization of chitin, which could reduce the molecular weight of the final product, alternative deproteinization strategies were attempted. Testing of the previously reported deproteinization method using aqueous K_3_PO_4_ resulted in protein reduction by factors varying from 2 to 10, but resulted in significant phosphate salt contamination of the final product. Contrarily, the incorporation of GRAS (Generally Recognized as Safe) compound Polysorbate 80 into the polymer washing step provided the polymer of comparable purity with no contaminants. This study presents new options for the deproteinization of chitin that can replace traditional approaches with methods that are environmentally friendly and can produce high purity polymer.

## 1. Introduction

**Figure 1:**
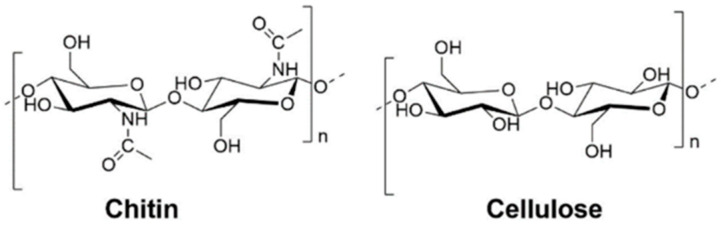
Chemical structure of chitin (**left**) and cellulose (**right**).

Chitin is one of the most abundant naturally occurring polysaccharides on Earth, second only to cellulose [1]. Structurally, it is very similar to cellulose except for an additional acetamide side chain at the C-2 position (Figure 1). Chitin polymer comprises repeating 2-(acetylamino)-2-deoxy-D-glucose *β*-linked units [2] and is naturally occurring in crustacean waste biomass (e.g., shrimp, lobster, and crabs), in amounts from 15–40% [3]. Additionally, crustacean biomass contains proteins in amounts from 20–40% and minerals from 20–50% [4,5].

The use of naturally occurring chitin as a substitute for plastic is widely considered beneficial for both the environment and the economy because of the ecological damage caused by petrochemical plastics [6]. Thus, chitin has shown potential for use as biomaterials (currently prepared from plastics [7]) due to its biocompatibility [8], which allows it to be used in a variety of medical devices such as sutures, wound healing gauges, drug delivery vehicles, and tissue engineering devices [6]. All these high-value materials, however, would need chitin of extremely high quality. Chitin obtained through the use of ionic liquids (ILs) [9], mostly IL 1-ethyl-3-methylimidazolium acetate ([C_2_mim][OAc]), was found to be the best type of chitin for many applications (e.g., electrospinning) due to its high molecular weight (MW) compared to commercial “pulped” sources [10,11,12].

However, in order for chitin to be used in medical applications, there must be a way to ensure this IL-extracted chitin meets the requirements normally associated with good manufacturing practice (GMP) to qualify as a ‘medical grade’ polymer [13]. This means the polymer should at least meet USP Class VI requirements, which refer to a series of biological tests performed on the material to demonstrate its safety [14]. Because chitin is isolated from sources of biological origin, such as crustacean shells, it might have trace amounts of proteins, which should be quantified; ideally, medical grade material should be completely free of proteins. The presence of trace proteins may have implications when used on humans [15]. According to one study, crustaceans cause allergic reaction is up to 2% of people in the United States [16]. These allergenic proteins include tropomyosin (TM), arginine kinase (AK), sarcoplasmic calcium-binding protein (SCP), and myosin light and heavy chain, depending on the species of crustacean [5]. TM has been demonstrated to be the most prevalent allergen present, and extended exposure to this protein can lead to upper and lower respiratory symptoms (asthma, rhinitis), skin conditions, and development of ingestion-related food allergies. Over 80% of shellfish-sensitized patients are known to react to this allergenic protein. Moreover, the use of elevated temperature alone does not deactivate its biological action: TM displays a remarkable resistance to heating, retaining its allergenicity even in heat-processed products obtained from shellfish [17], making it even harder to remove. Since TM and these other proteins are found in shells, they likely remain in chitin throughout the isolation process.

At the same time, contrarily to pulped chitin (‘practical grade chitin’ or PG-chitin), which is demineralized with HCl and deproteinized with NaOH during its isolation, chitin obtained through ‘microwave-assisted extraction’ using [C_2_mim][OAc] is not additionally deproteinized [11]. As the presence of proteins is inevitable when working with biomass of animal origin regardless of the isolation method employed, it is necessary to determine the purity of the resultant polymer and quantify residual protein content in extracted chitin. In prior attempts to quantify proteins, a method was demonstrated for the determination of the polymer purity using solid-state cross-polarization magic angle spinning carbon-13 nuclear magnetic resonance (SS CP MAS ^13^C NMR) spectroscopy [18]. However, the exact amount of proteins present in shrimp shells could not be determined by this method, as it resulted in the quantification of impurities altogether, not just proteins.

If proteinaceous materials are present in the polymer, their removal can be difficult [19,20]. While ‘traditional’ deproteinization methods using bases [21,22] work well to purify PG-chitin obtained by pulping [11], they cannot be employed on high MW polymers intended for the manufacture of medical products. Harsh chemicals (i.e., those traditionally used for deproteinization) would alter the polymer’s MW [14,18,23], thus they cannot be used on IL-chitin without sacrificing its properties [11,12,23], and hazardous chemicals would defeat the rationale of the polymer use for medical applications. Other deproteinization techniques employed during the chitin isolation processes include quite slow enzymatic and microbiological methods [24,25,26].

In the search for proper deproteinization agents, potassium phosphate (K_3_PO_4_) was found to be used for the deproteinization of bile and plasma samples in the pharmacokinetic study of cyclosporin A and ketoconazole [27]. Based on the reported work, the deproteinization of chitin polymer using aqueous solutions of K_3_PO_4_ (hereafter abbreviated K_3_PO_4aq_) was attempted by Rogers’ group [28]. This deproteinization strategy employed coagulation of the chitin/[C_2_mim][OAc] solution into K_3_PO_4aq_, forming an aqueous three-component biphasic system (ABS) [28]. At the same time, the cost driver for the chitin extraction is the recovery and re-use of the IL, and the formation of ABS might significantly complicate the IL recycle.

In order to implement deproteinization at the end, or ‘downstream’ process, K_3_PO_4aq_ washings after completion of the IL removal step were included, and proteins not previously properly documented were quantified. It was also hypothesized that the use of surfactants (ideally from the Generally Recognized as Safe (GRAS) list, and inexpensive) for the deproteinization would be a much better option than the use of relatively expensive, low solubility and difficult-to-remove-from-chitin phosphate salt.

Polysorbate 80 (Tween^®^ 80), a GRAS compound, which is included in the United States Pharmacopeia, the National Formulary list (USP/NF) [29,30], British Pharmacopoeia (BP) list [31], and European Pharmacopoeia (EP) list [32], was chosen as an approved pharmaceutical excipient for use in oral active pharmaceutical ingredient (API) formulations. It is generally regarded as a non-toxic and non-irritating, with an acceptable daily intake (ADI) of 25 mg/kg body weight [33]. In this communication, proteins in ‘IL-extracted chitin’ were identified and quantified. The use of aqueous solutions of K_3_PO_4_ and Tween^®^ 80 (Polysorbate 80) for chitin deproteinization ‘downstream’ of the IL-chitin extraction process was studied.

## 2. Results and Discussion

### 2.1. Characterization of IL-Extracted Chitin (IL-Chitin) and Determination of the Amount of Proteins

Initial determination of the amount of proteins in chitin involved testing IL-chitin. To prepare IL-chitin, shrimp shell biomass (ESI, Figure S1a) was first characterized; the chitin content of this biomass was determined to be 18 ± 2% using the Black and Schwartz method [19]. The biomass was washed with DI water, oven-dried, ground, sieved, and subjected to microwave-assisted extraction using [C_2_mim][OAc] as previously reported [34], with small modifications to the overall duration of heating and microwave pulsing (see Materials and Methods Section).

After microwave-assisted extraction of chitin, the solution was centrifuged, and coagulated using a ‘standard’ coagulation and washing technique, also as previously reported [34]. During the coagulation of the chitin-IL solution using water, water competes with the chitin-IL interactions and promotes chitin precipitation. Precipitated chitin (in the form of swollen gel) was thoroughly washed with water from [C_2_mim][OAc] solvent.

**Figure 2:**
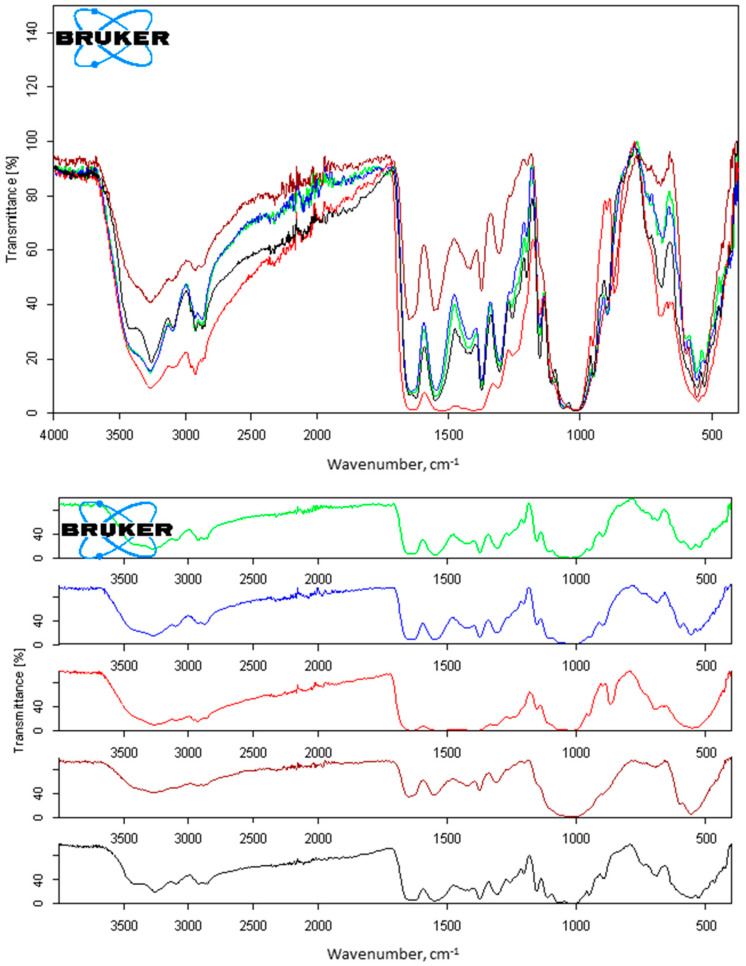
FTIR spectra of chitin materials obtained utilizing K_3_PO_4aq_ for deproteinization. Red: shrimp shell biomass, black: IL-chitin; lime green: IL-chitin_dry/K3PO4_; blue: IL-chitin_swollen/K_3___PO_4__; burgundy: chitin obtained in accordance with reference, by coagulation of IL-chitin solution into K_3_PO_4_ instead of water [28]. **Top**: normalized spectra; **bottom**: stacked spectra.

After subsequent drying, this IL-chitin appeared as a dark brown powder (ESI, Figure S1b)). The powder was ground and characterized using Fourier transform infrared (FTIR) spectroscopy and powder X-ray diffraction (pXRD). The FTIR spectrum (Figure 2, black trace) displayed patterns that are typical for IL-chitin [35,36,37,38]: the O-H stretching located at 3432 and 3096 cm^−1^; C-H stretches of CH, CH_2_, and CH_3_ groups of chitin at 2927 cm^−1^, 2915 cm^−l^, and 2876 cm^−l^, respectively; vibration modes of amide I in the region 1660–1620 cm^−1^, which appears in the form of two peaks, at 1648 cm^−1^ and 1622 cm^−1^; C-N + N-H (Amide III) at 1303 cm^−1^, and asymmetric N-H at 3260 cm^−1^. Multiple peaks were also seen within the region 1020–1160 cm^−1^ and were associated with vibration modes of C-OH bonds. Asymmetric bridge stretching (C-O-C ring) was located at 1152 cm^−1^.

Similarly, the pXRD diffractogram (ESI, Figure S2, black trace) showed peaks present at 9.0°, 19.3°, and 26.8° 2θ attributed to the chitin polymer. The pXRD analysis did not display any peaks corresponding to calcium carbonate (CaCO_3_) at 29.6° 2θ or calcium phosphate (Ca_3_(PO_4_)_2_) at 32.0° 2θ, showing that all minerals were fully removed through centrifugation.

The total protein content of the IL-chitin was attained through an Advanced Protein Assay (APA). The reagent used for APA combines the advantages of its ability to determine low protein content through signal sensitivity, and its suitability for proteins’ variability. A simple, one step procedure enables the calculation of the protein content of a sample by observing the optical density using a spectrophotometer and then calculating the amount of the protein present in the sample using defined variables such as cell geometry and sample concentration. This test has been demonstrated as a useful tool for quantifying the amount of protein in chitin samples. The obtained IL-chitin was determined to have an overall protein content of 15,882 ± 3422 µg/g (Table 1). As a benchmark, we also used commercial PG-chitin (obtained by pulping shrimp biomass with HCl and NaOH) and, thus, deproteinized with NaOH. To ensure similar treatment conditions, this chitin was also dried, ground, and sieved to less than 125 µm particle size. Expectedly, the level of proteins in commercial chitin was below the detection limit of the technique, <1 µg/mL.

Enzyme-Linked Immunosorbent Assay (ELISA), a plate-based assay technique was employed to quantify the amount of TM present in the IL-chitin. The ELISA plates were pre-coated with anti-tropomyosin monoclonal antibody 1A6, immobilized on a solid surface, which binds to specific epitope present on *D. pteronyssinus* tropomyosin allergen, Der p 10 [39]. Following incubations with IL-chitin extract and a secondary detection antibody, TM concentration was determined using a calibration curve generated from purified TM allergen. Through this test, it was observed that IL-chitin contained 0.3175 ± 0.1475 µg/g of tropomyosin. Mass spectrometry (MS) has been used for the identification of proteins. It was found that chitin contained 152 proteins in 108 clusters including allergenic tropomyosin, skeletal muscle actin, and a cluster of myosin light and heavy chain types 1 and 2. The complete protein list is provided in ESI.

### 2.2. Treatment of IL-Extracted Chitin (IL-Chitin) with Aqueous K_3_PO_4_

#### 2.2.1. Washing IL-Extracted Chitin with K_3_PO_4aq_ in a Dry State

Once the protein content of IL-chitin was determined, the air-dried polymer ‘as prepared’ was washed with K_3_PO_4aq_, to determine if this treatment would result in complete removal of proteins. The resulting chitin was abbreviated as IL-chitin_dry/K_3___PO_4__ (where ‘dry’ indicates that the polymer was washed after drying and K_3_PO_4_ was used as a deproteinization agent). The washings were carried out using 40 wt% aqueous solution of K_3_PO_4_, at room temperature. The IL-chitin to washing reagent ratio was 1/25 g/g. The mixture of IL-chitin and K_3_PO_4aq_ was stirred using a magnetic stirrer, after which IL-chitin was filtered, washed with fresh DI water on the filter, and dried. The color of IL-chitin_dry/K_3___PO_4__ was slightly lighter than prior to washing (ESI, Figure S1e).

The washed IL-chitin_dry/K_3___PO_4__ was again characterized using FTIR (Figure 2, lime green trace) and pXRD (see ESI, Figure S2, lime green trace). Few differences with FTIR spectra of IL-chitin were found in the position and/or intensity of the main peaks. Thus, the O-H stretching at 3432 cm^−1^ became significantly more pronounced, indicating less hydrogen bonding in the ‘washed’ polymer. Secondly, IL-chitin demonstrated two separate, clearly distinguishable peaks at 1648 cm^−1^ and 1622 cm^−1^ for amide I due to two types of carbonyl in amide moiety: those that are hydrogen-bonded to the amino group inside the same chain (C=O...H-N) at 1648 cm^−1^, and those hydrogen-bonded to C(6)OH from the side chain (-CH_2_OH…O=C), at 1622 cm^−1^. Contrarily, chitin that was additionally washed with K_3_PO_4_ revealed a converging of these peaks into a single band at 1642 cm^−1^, implying partial amorphization [40]. Finally, peaks associated with vibration modes of C-OH, C-O-C, and C-C bonds overlapped with stretching and bending vibrations of phosphate ion [41], overall making this region significantly more intense.

Confirming FTIR data, the pXRD diffractogram also indicated the presence of K_3_PO_4_ hydrates (K_3_PO_4_ ·1.5H_2_O, K_3_PO_4_ ·3H_2_O, and K_3_PO_4_ ·7H_2_O [41]) with an unknown ratio between them, as an assembly of peaks at ~30–35° 2θ).

This was not entirely unexpected because a previously reported method of the *coagulation* of IL-chitin in K_3_PO_4_ [28] in place of water resulted in a significant amount of salt remaining in the polymer, even after multiple washing steps. To be certain of such contamination, the experiment was repeated as previously published [28], and the obtained chitin was characterized using FTIR (Figure 2, burgundy trace) and pXRD (ESI, Figure S2, burgundy trace). The FTIR spectrum and pXRD diffractogram of chitin coagulated in K_3_PO_4_ solution showed significant contamination of chitin with K_3_PO_4_.

The total protein content of the IL-chitin_dry/K3PO4_ obtained through APA was found to be 6,990 ± 453 µg/g (Table 1). The protein concentration was still relatively high, although this simple washing decreased the protein amount almost two-fold.

#### 2.2.2. Washing IL-Extracted Chitin with K_3_PO_4aq_ in a Swollen State

It is worth noting first that when chitin polymer is dissolved in the IL, its hydrogen-bonding network is fully destroyed, thus providing an amorphous chitin with a low degree of crystallinity, making the polymer chain hydrated and fully accessible to the reactant(s). This particular property of chitin was utilized in the next experiment. After the microwave-assisted extraction, centrifugation of minerals, coagulation in water, and proper washing of chitin from the IL, a portion of water-swollen polymer gel was subjected to washing with K_3_PO_4 aq_. After this, the chitin was again washed with water, in an attempt to remove K_3_PO_4_ that could be trapped within the polymeric network, to obtain IL-chitin_swollen/K3PO4_. The color was significantly lighter than either IL-chitin or IL-chitin_dry/K3PO4_ (ESI, Figure S1c). However, even though multiple water washings were conducted, a significant amount of K_3_PO_4_ appeared to remain in the IL-chitin_swollen/K3PO4_ polymer, as could be seen in its FTIR spectrum (Figure 2, blue trace). The amount of K_3_PO_4_ remaining in the IL-chitin_swollen/K3PO4_ was greater than that after a washing of the polymer in the dry state, although less than in the case of the coagulation of the IL-chitin solution in K_3_PO_4aq_. pXRD diffractogram (see ESI, Figure S2, blue trace) confirmed the FTIR observations. Similar to the previous experiment, APA analysis was conducted, and it was found that the overall protein content of the polymer was 1398 µg/g, ca. a 10-fold decrease in protein content when compared to untreated material (Table 1). Despite a significant success in the removal of proteins, the chitin was highly contaminated with K_3_PO_4_ due to the likely complexation of salt with the polymer.

### 2.3. Treatment of IL-Extracted Chitin (IL-Chitin) with Aqueous Tween^®^ 80

#### 2.3.1. Washing IL-Extracted Chitin with Aqueous Tween^®^ 80 in Dry State

Considering that washing of chitin with K_3_PO_4aq_ resulted in trapping phosphate salt within chitin’s polymeric network, GRAS-list water-soluble liquid surfactants were attempted for deproteinization. Polysorbate 80 (Tween^®^ 80), was dissolved in water (10 wt%) to prepare an aqueous solution. First, similar to the experiments with K_3_PO_4aq_ described above, the polymer was extracted with the help of IL, coagulated and washed using water, and then air-dried.

**Figure 3:**
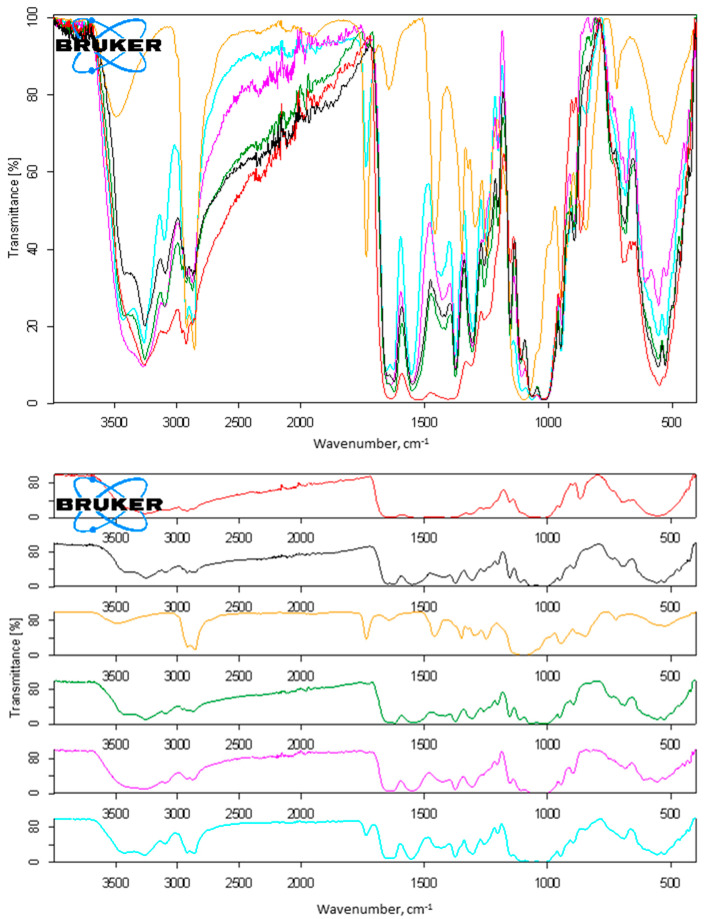
FTIR from chitin utilizing polysorbate 80 for deproteinization (red: shrimp shell biomass; orange: Polysorbate 80; black: IL-chitin; dark green: IL-chitin_dry/Polysorbate 80_; pink: IL-chitin_swollen/Polysorbate 80_; aqua: chitin obtained by coagulation of IL-chitin solution into Polysorbate 80 instead of water. **Top**: normalized spectra; **bottom**: stacked spectra.

The polymer was washed with Tween^®^ 80 for 12 h on a stirring plate, after which the IL-chitin was filtered, washed with fresh DI water on the filter, and dried. The color of the IL-chitin_dry/Polysorbate 80_ did not significantly change after washing (ESI, Figure S1f). The FTIR spectrum (Figure 3, dark green trace) appeared to be very similar to that of untreated chitin, with the only exception of a somewhat more pronounced O-H at 3410 and 3096 cm^−1^, indicating the weakening of the polymer’s hydrogen bonding. The pXRD diffractogram (ESI, Figure S3, dark green trace) also did not reveal significant differences between IL-chitin and IL-chitin_dry/Polysorbate 80_. However, this sample was not subjected to APA analysis due to its relatively dark color, which, as was previously determined, was associated with the presence of a high amount of proteins.

#### 2.3.2. Washing IL-Extracted Chitin with Aqueous Polysorbate-80 (Tween^®^ 80) in Swollen State

Following this experiment, after microwave-assisted extraction, centrifugation of minerals, coagulation in water, and proper washing of chitin from the IL, a portion of the water-swollen polymer gel was subjected to washing with aqueous Tween^®^ 80. Two coagulation approaches were investigated: (a) ‘standard’ coagulation followed by the Tween^®^ 80 wash of a swollen gel and then water wash, and (b) coagulation of the IL-chitin solution into the solution of Tween^®^ 80 followed by a water wash, both in a manner similar to the experiments with phosphate, although a larger number of washing steps were required. After proper washing, the polymer was dried overnight, producing an off-white solid in both cases, drastically different in color than ‘untreated’ chitin (ESI, Figure S1d,g).

Both solids were subjected to characterization. The FTIR spectra of IL-chitin_swollen/Polysorbate 80_ conformed to the chitin standard displaying peaks of pure chitin polymer (Figure 3, pink trace), with few subtle changes. Thus, a more pronounced O-H peak was located at 3411 cm^−1^, implying weaker hydrogen bonding for the Polysorbate-washed polymer. Amide II peaks at 1648 cm^−1^ and 1622 cm^−1^ converged into a single band at 1642 cm^−1^, suggesting a partial amorphization, similarly to the polymer that was washed with the K_3_PO_4_-solution in a swollen state. The peaks associated with the vibration modes of C-OH and C-O-C insignificantly decreased. No Polysorbate 80 peaks were detected (the spectrum of Polysorbate 80 is also provided in Figure 3, orange trace).

Crystallographic pXRD patterns also conformed to the standard. Moreover, IL-chitin_swollen/Polysorbate 80_ displayed no traces of minerals in pXRD (ESI, Figure S3, pink trace), thus presenting a significant advantage when comparing with the K_3_PO_4aq_ wash. This may be the result of the polysorbate forming complexes with ionic minerals such as potassium and calcium [42]. APA analysis showed that the amount of proteins decreased almost 20 times after washing the swollen chitin with Tween^®^ 80 followed by water washing (1110 ± 90 (0.11%), Table 2).

The coagulation of the IL-chitin solution into the solution of Tween^®^ 80 followed by the water wash, in a manner similar to the experiments with phosphate, resulted in chitin contaminated with polysorbate (Figure 2, aqua trace), which can be seen by the clearly identifiable Polysorbate 80 peaks at 1735, 1637, 1461, and 844 cm^−1^. No differences with IL-chitin were seen in pXRD (ESI, Figure S3, aqua trace), except that the polymer appeared much more amorphous. It does appear that the coagulation of the IL-chitin solution must be conducted in pure water, otherwise deproteinization agents become trapped in the polymer and this results in the polymer’s contamination. No APA analysis was conducted for this sample.

## 3. Materials and Methods

### 3.1. Materials for Chitin Extraction and Coagulation

The IL, 1-ethyl-3-methylimidazolium acetate ([C_2_mim][OAc], purity > 95%), was purchased from ProIonic (Grambach, Austria). The potassium phosphate (K_3_PO_4_, tribasic anhydrous) was purchased from VWR (Radnor, PA, USA). The Tween^®^ 80 (Polysorbate 80) was purchased from Traverse Bay Bath and Body (Traverse City, MI, USA). Deionized (DI) water used for the coagulation and washing of chitin was purified using Evoqua Water Technologies system LDIRS03 (Richmond, VA, USA). Crustacean biomass (shrimp shells, 18% chitin content) was ground and sieved to <125 µm and then pre-dried in 50 °C oven overnight.

### 3.2. Materials for Biochemical Studies

Advanced Protein Assay Reagent was obtained from Cytoskeleton (ADV01, Denver, CO, USA). Tropomyosin ELISA kit (EL-TPM) was obtained from InBio (Charlottesville, VA, USA).

### 3.3. Laboratory Processing of Dry Shrimp Shells

The dried shrimp shells were ground by use of an electric lab mill (Model M20 S3, Ika^®^, Wilmington, NC). The ground shrimp shells were sieved through a stainless-steel mesh (Gilson Co., Lewis Center, OH, USA) in order to obtain shrimp shell particles with diameters less than 250 µm. Finally, the sieved particles were dried at 90 °C overnight in an oven (SKU# 52412-83, Cole-Parmer Instrument Co., Vernon Hills, IL, USA).

### 3.4. Extraction of Chitin Using Microwave Irradiation

Chitin was extracted from dry, ground, and sieved shrimp shells using a microwave-assisted extraction procedure [9]. Briefly, 4 g of shrimp shells and 196 g of the IL were mixed in a 250 mL Erlenmeyer flask. The two components were stirred together until all biomass was suspended in the IL and heated by use of short microwave pulses (Sunbeam Products, Model SGB8901, Boca Raton, FL, USA). The length of each pulse was 2 s for the first minute and 3 s for the next 5 min; in between sets of 3 pulses, the reaction mixture was stirred manually for few seconds to allow even heat distribution. Afterwards, the hot reaction mixture was centrifuged (Thermo Scientific, Sorvall Legend XTR Centrifuge, Waltham, MA, USA) at 3800 rpm for 10 min to collect undissolved shrimp shell residues at the bottom of the centrifuge tubes, allowing removal from the chitin extract.

The IL-chitin extract obtained from the aforementioned procedure was coagulated by slowly pouring the extract into approximately 500 mL of water while being stirred at 500 rpm on a stir plate (IKA, RCT Basic, Wilmington, NC, USA). In about 1 h, the mixture was poured through a 90 µm stainless-steel sieve to allow removal of IL and water. The remaining solid was then transferred into approximately 500 mL of fresh DI water, and after 1 h was again poured through a 90 µm stainless-steel sieve. This was repeated 13 times. At the end of 14 washes, the solid was transferred into a crystallization dish lined with parchment paper and allowed to dry overnight in an oven at 90 °C.

### 3.5. Washing Chitin Using K_3_PO_4aq_

#### 3.5.1. Washing Dry Chitin

Dry, ground chitin obtained through IL extraction (2 g) was placed into 40 wt% K_3_PO_4aq_ (50 g) in 100 mL beaker. The suspension was stirred for 5 days using magnetic stirrer (IKA, RCT Basic, Wilmington, NC, USA). Then, chitin was filtered using a fritted filtering funnel (M porosity Quark Glass, Vineland, NJ, USA) and thoroughly washed with water. After washing, chitin was dried in the oven (StableTemp, SKU# 52412-83, Cole-Parmer Instrument Co., Vernon Hills, IL, USA).

#### 3.5.2. Washing Swollen Chitin

The IL-chitin mixture was slowly poured into 500 mL of water while being stirred at 500 rpm on a magnetic stirrer. Then, every hour for 5 h, the mixture was sieved through a 90 µm stainless-steel mesh to remove IL and water. The remaining solid was then transferred into 500 mL of fresh DI water. The solid was then sieved and transferred into K_3_PO_4 aq_ (340 g of 25 wt% aqueous solution) in a 600 mL beaker. After the system equilibrated for 1 h, the solid was sieved through a 90 µm stainless-steel and then placed into fresh K_3_PO_4aq_ (340 g of 25 wt% aqueous solution), for the second wash. After an additional 1 h of equilibrating, the solid was washed with fresh DI water 6 times using the ‘standard method’ (500 mL of DI water each time). At the end of washing, the solid was transferred into a crystallization dish lined with parchment paper and allowed to dry overnight in the oven at 90 °C.

#### 3.5.3. Coagulating IL-Chitin Solution in K_3_PO_4aq_

This experiment was conducted in accordance with [28], only to compare the amount of residual K_3_PO_4_ in the polymer. In brief, 680 g of 25 wt% K_3_PO_4aq_ was added to a 1000 mL beaker, and IL-chitin mixture was slowly poured into the beaker while stirring with stir bar at 500 rpm. Then, the mixture was equilibrated overnight for IL to leach out of the coagulated polymer. The polymer was washed in the same manner as the polymer coagulated in water, where every hour the mixture was poured through a 250 µm sieve and the solid was transferred into approximately 500 mL of fresh DI water. At the end of 14 washes, the solid was transferred into a crystallization dish lined with parchment paper and allowed to dry overnight in the oven at 90 °C.

### 3.6. Washing Chitin Using Tween^®^ 80

#### 3.6.1. Washing Dry Chitin

Dry, ground chitin obtained through IL-extraction (2 g) was placed into Tween^®^ 80 (Polysorbate 80) solution, 10 wt.% in water. The suspension was stirred for 12 h using a magnetic stirrer (IKA, RCT Basic, Wilmington, NC, USA). After that, chitin was filtered using a fritted filtering funnel (M porosity Quark Glass, Vineland, NJ, USA), and thoroughly washed with water. After washing, chitin was dried in the oven (StableTemp, SKU# 52412-83, Cole-Parmer Instrument Co., Vernon Hills, IL, USA).

#### 3.6.2. Washing Swollen Chitin with Tween^®^ 80

The IL-chitin mixture was slowly poured into 500 mL of water while being stirred at 500 rpm on a magnetic stirrer. Then, every hour for 5 h, the mixture was sieved through a 90 µm stainless-steel mesh to remove IL and water. The remaining solid was transferred into 500 mL of fresh DI water. The solid was then sieved and transferred into Tween 80 (200 g of 10 wt% aqueous solution) in a 600 mL beaker. After the system equilibrated for 1 h, the solid was sieved through a 90 µm stainless-steel mesh and then placed into fresh Tween 80 (200 g of 10 wt% aqueous solution), for the second wash. After another hour of equilibrating, the solid was washed with fresh DI water 10 times using the ‘standard method’ (200 mL of DI water each time). At the end of washing, the solid was transferred into a crystallization dish lined with parchment paper and allowed to dry overnight in the oven at 90 °C.

#### 3.6.3. Coagulating IL-Chitin Solution in Tween^®^ 80

Solution of K_3_PO_4_ (200 g of 10 wt% K_3_PO_4aq_) was added to a 600 mL beaker, and IL-chitin mixture (60 g) was slowly poured into the beaker while stirring with stir bar at 500 rpm. Then, the mixture was equilibrated overnight for IL to leach out of the coagulated polymer. The polymer was washed in the same manner as the polymer coagulated in water, where every hour the mixture was poured through a 250 µm sieve and the solid was transferred into approximately 500 mL of fresh DI water. At the end of 14 washes, the solid was transferred into a crystallization dish lined with parchment paper and allowed to dry overnight in the oven at 90 °C.

### 3.7. Fourier Transform Infrared Spectroscopy (FTIR)

Chitin products were characterized as ground powder (<125 µm) by use of Bruker Alpha FTIR instrument (Bruker Optics Inc., Billerica, MA, USA). FTIR Spectra were recorded in the transmission mode, in the range of 4000–400 cm^−1^, with 64 scans at room temperature.

### 3.8. Powder X-ray Diffraction (pXRD)

pXRD diffractograms of ground samples (<125 µm) were obtained in the range of 2θ 2–40° using a Bruker D2 Phaser diffractometer (Bruker AXS, Inc., Ewing Township, NJ, USA).

### 3.9. Spectrophotometer/Optical Density Readings

Spectrophotometer readings were performed using Implen NanoPhotometer™ P-Class Spectrophotometer (Munich, Germany).

### 3.10. Tropomyosin Quantification

Tropomyosin was quantified using an ELISA tropomyosin kit from InBio. A polystyrene microtiter plate was coated with mAb 1A6 (100 uL/well) in 50 mM carbonate–bicarbonate buffer (pH 9.6), and refrigerated overnight at 4 °C.

The plate was washed 3 times with phosphate-buffered saline, pH 7.4 containing 0.05% Tween^®^ 20 (PBS-T). Tropomyosin allergen standard and chitin extract samples were added to the plate and incubated for 1 h at room temperature. The plate was washed as previously described, followed by addition of polyclonal detection antibody and 1-h incubation at room temperature. The plate was washed, followed by addition of peroxidase-conjugated goat anti-rabbit antibody and 30-min incubation at room temperature. Following a final plate wash, ABTS developing substrate was added. Optical density (OD) was measured at 405 nm. Tropomyosin concentration in chitin extracts was determined by comparison to the TM standard curve.

### 3.11. Quantification of Total Protein Content Using APA

Chitin extracts were prepared by adding chitin samples to PBS-T (100 mg/mL) and vortexing for 30 s. Extraction continued with gentle agitation on a rocking platform for 2 h at room temperature. Samples were centrifuged at 2000× *g* to allow separation of the protein extract from chitin solids.

APA Buffer was prepared by mixing 4 mL of deionized water and 1 mL of Advanced Protein Assay Reagent (Cytoskeleton Inc., Cat. No. ADV01). For protein quantification, ready APA Buffer (1 mL) was mixed with 10 µL of the chitin extract in an Eppendorf tube and thoroughly mixed by gentle inversion. Similarly, a ‘blank’ was prepared by mixing APA buffer (1 mL) with 10 µL of PBS-T in an Eppendorf tube and mixed by gentle inversion. The samples and ‘blank’ were transferred to a cuvette with 1 cm pathlength. A spectrophotometer (Implen NanoPhotometer™ P-Class, Munich, Germany) was used to measure the OD at 590 nm of the blank sample, which was subtracted from the OD values measured for chitin samples. Protein concentration was determined using the formula OD_590_ = 30 µg protein/mL reagent/cm light pathlength.

### 3.12. Mass Spectrometry

The mass spectrometry instrument used to analyze the samples was a Xevo G2 QTof coupled to a nanoAcquity UPLC system (Waters, Milford, MA). Samples were loaded onto a C18 Waters Trizaic nanotile of 85 µm × 100 mm; 1.7 μm (Waters, Milford, MA, USA). The column temperature was set to 45 °C with a flow rate of 0.45 mL/min. The mobile phase consisted of A (water containing 0.1% formic acid) and B (acetonitrile containing 0.1% formic acid). A linear gradient elution program was used: 0–40 min, 3–40% (B); 40–42 min, 40–85% (B); 42–46 min, 85% (B); 46–48 min, 85–3% (B); 48–60 min, 3% (B).

Mass spectrometry data were recorded for 60 min for each run and controlled by MassLynx 4.1 (Waters, Milford, MA, USA). The acquisition mode was set to positive polarity under resolution mode. The mass range was set from 50–2000 Da. The capillary voltage was 3.5 kV, sampling cone at 25 V, and extraction cone at 2.5 V. The source temperature was held at 110 °C. The cone gas was set to 25 L/h, nano flow gas at 0.10 Bar, and desolvation gas at 1200 L/h. Leucine–enkephalin at 720 pmol/uL (Waters, Milford, MA, USA) was used as the lock mass ion at *m*/*z* 556.2771 and introduced at 1 uL/min at 45 s intervals with a 3-scan average and mass window of +/− 0.5 Da. The MSe data were acquired using two scan functions corresponding to low energy for function 1 and high energy for function 2. Function 1 had collision energy at 6 V and function 2 had a collision energy ramp of 18–42 V.

RAW MSe files were processed using Protein Lynx Global Server (PLGS) version 2.5.3 (Waters, Milford, MA, USA). Processing parameters consisted of a low energy threshold set at 200.0 counts, an elevated energy threshold set at 25.0 counts, and an intensity threshold set at 1500 counts. The databank used was constructed from the known sequence of the protein of interest plus other random proteins as well as common contaminants including human keratins, porcine trypsin, bovine serum albumin, bovine beta-casein, as well as their randomized sequences.

Searches were performed with trypsin specificity and allowed for three missed cleavages. Possible structure modifications included for consideration were methionine oxidation and carbamidomethylation of cysteine. For viewing, PLGS search results were exported in Scaffold v4.4.6 (Proteome Software Inc., Portland, OR, USA).

Scaffold (version Scaffold_4.8.9, Proteome Software Inc., Portland, OR, USA) was used to validate MS/MS-based peptide and protein identifications. Peptide identifications were accepted if they could be established at greater than 20.0% probability by the Scaffold Local FDR algorithm. Protein identifications were accepted if they could be established at greater than 20.0% probability and contained at least 1 identified peptide. Protein probabilities were assigned by the Protein Prophet algorithm [43]. Proteins that contained similar peptides and could not be differentiated based on MS/MS analysis alone were grouped to satisfy the principles of parsimony. Proteins sharing significant peptide evidence were grouped into clusters.

## 4. Conclusions

Chitin obtained through microwave-assisted extraction using [C_2_mim][OAc] IL is not additionally deproteinized. The protein contaminants were identified, and allergenic proteins such as tropomyosin, skeletal muscle actin, and cluster of myosin light and heavy chain types 1 and 2 were detected. The overall amount of proteins present in this type of chitin was significant and varied from 1.3 to 1.9 wt%.

Washing the air-dried polymer ‘downstream’ of the IL-chitin extraction process with aqueous K_3_PO_4_ solution decreased the protein level ~2 times (to 0.7 wt%), while washing water-swollen polymer gel decreased the amount of proteins ~10 times (to 0.14 wt%), possibly due to the higher accessibility of polymer chains to the washing reagent. At the same time, the use of aqueous K_3_PO_4_ solution resulted in the contamination of the chitin polymer with K_3_PO_4_ salt. Alternatively, using water-soluble GRAS chemical Tween^®^ 80 (Polysorbate 80) for chitin deproteinization resulted in the polymer of a similar protein contamination (0.11 wt% of the proteins) but with much higher polymer purity because of the complete removal of residual minerals.

This study presents new options for the deproteinization of chitin that can replace traditional approaches with methods that are environmentally friendly and can produce high purity polymer. This method supports innovative and environmentally-aware research and development efforts focused toward developing and sustaining future industrial processes and products based on positive environmental and economic advances.

## Figures and Tables

**Table 1:** Treatment of IL-chitin (wet and gel form) with K_3_PO_4_ and APA analyses results.

Type of Chitin	Treatment	Protein Content Determined through APA, µg/g (%)
IL-chitin	1. Extraction with [C_2_mim][OAc]; 2. coagulation in DI water; 3. washing with DI water; 4. drying	15,882 ± 3422 (1.59%)
PG-chitin	Commercial, no additional chemical treatment employed	<1
IL-chitin_dry/K3PO4_	1. Extraction with [C_2_mim][OAc]; 2. coagulation in DI water; 3. washing with DI water; 4. drying; 5. washing dry chitin with 40 wt% K_3_PO_4aq_; 6. washing with DI water; 7. drying	6990 ± 453 (0.70%)
IL-chitin_swollen/K3PO4_	1. Extraction with [C_2_mim][OAc]; 2. coagulation in DI water; 3. washing with DI water; 4. washing (x2) swollen gel chitin with 25 wt% K_3_PO_4aq_; 5. washing with DI water; 6. drying	1398 ± 256 (0.14%)

**Table 2:** Treatment of IL-chitin (wet and gel form) with Polysorbate 80 and APA analyses results.

Type of Chitin	Treatment	Protein Content Determined through APA, µg/g (%)
IL-chitin	1.Extraction with [C_2_mim][OAc]; 2. coagulation in DI water; 3. washing with DI water; 4. drying	15,882 ± 3422 (1.59%)
IL-chitin_swollen/Polysorbate 80_	1.Extraction with [C_2_mim][OAc]; 2. coagulation in water; 3. washing with Polysorbate 80; 4. washing with DI water, 5. drying	1110 ± 256 (0.11%)
IL-chitin_coagulated/Polysorbate 80_	1.Extraction with [C_2_mim][OAc]; 2. coagulation in Polysorbate 80; 3. washing with DI water, 4. drying	ND

## Data Availability

Not applicable.

## Supplementary Materials

The following supporting information can be downloaded at: https://www.mdpi.com/article/10.3390/molecules27133983/s1, Figure S1: (a) Biomass and (b) IL-chitin compared with chitin after washing in swollen state; in dry state, and coagulated in solutions of deproteinization agents; Figure S2: pXRD of chitin materials obtained utilizing K_3_PO_4 aq_ for deproteinization; Figure S3: pXRD from chitin utilizing Polysorbate 80 for deproteinization.
