# Supplementary material for: Deproteinization of Chitin Extracted with the Help of Ionic Liquids
Source: Molecules. 2022 Jun 21;27(13):3983. doi: 10.3390/molecules27133983 (PMC9268416; doi:10.3390/molecules27133983)
Supplement: Supplementary file 1 [file molecules-27-03983-s001.zip › molecules-1776872-supplementary.pdf]

# Electronic Supporting Information

## Deproteinization of Chitin Extracted with the Help of Ionic Liquids

Douglas R. Lyon, Jr,[1] Bryan R. Smith,[2] Noureddine Abidi,[3] and Julia L. Shamshina[3,*]

[1] Lummus Technology, Houston, TX 77086

[2] InBio, Charlottesville, VA 22903

[3] Fiber and Biopolymer Research Institute, Texas Tech University, Lubbock, TX 79409, USA

**Table of Contents**

***Chitin Appearance*** ........................................ **2**

***FTIR from $K_3PO_4$*** ........................................ *Error! Bookmark not defined.*

***pXRD from $K_3PO_4$*** ........................................ **4**

***FTIR from Polysorbate 80*** ........................................ *Error! Bookmark not defined.*

***pXRD from Polysorbate 80*** ........................................ **6**

**Chitin Appearance**

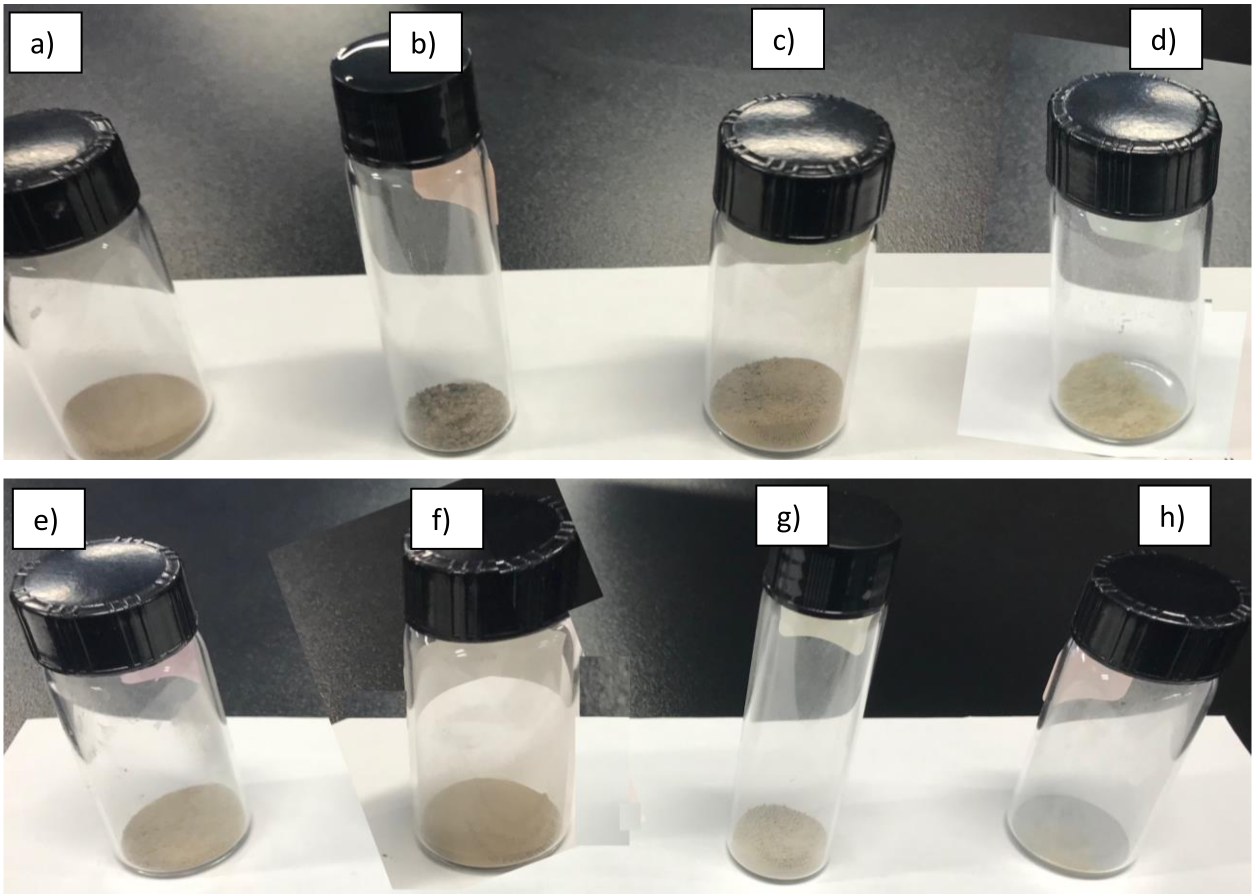


**Figure S1.** a) Biomass and b) IL-chitin compared with chitin after washing **in swollen state** (c) IL-chitin$_{swollen/K3PO4}$, d) IL-chitin$_{swollen/Polysorbate\ 80}$); **in dry state** (e) IL-chitin$_{dry/K3PO4}$, f) IL-chitin$_{dry/Polysorbate\ 80}$), and **coagulated in solutions** of deproteinization agents (g) IL-chitin$_{coag/K3PO4}$, h) IL-chitin$_{coag/Polysorbate\ 80}$).

**pXRD from $K_3PO_4$**

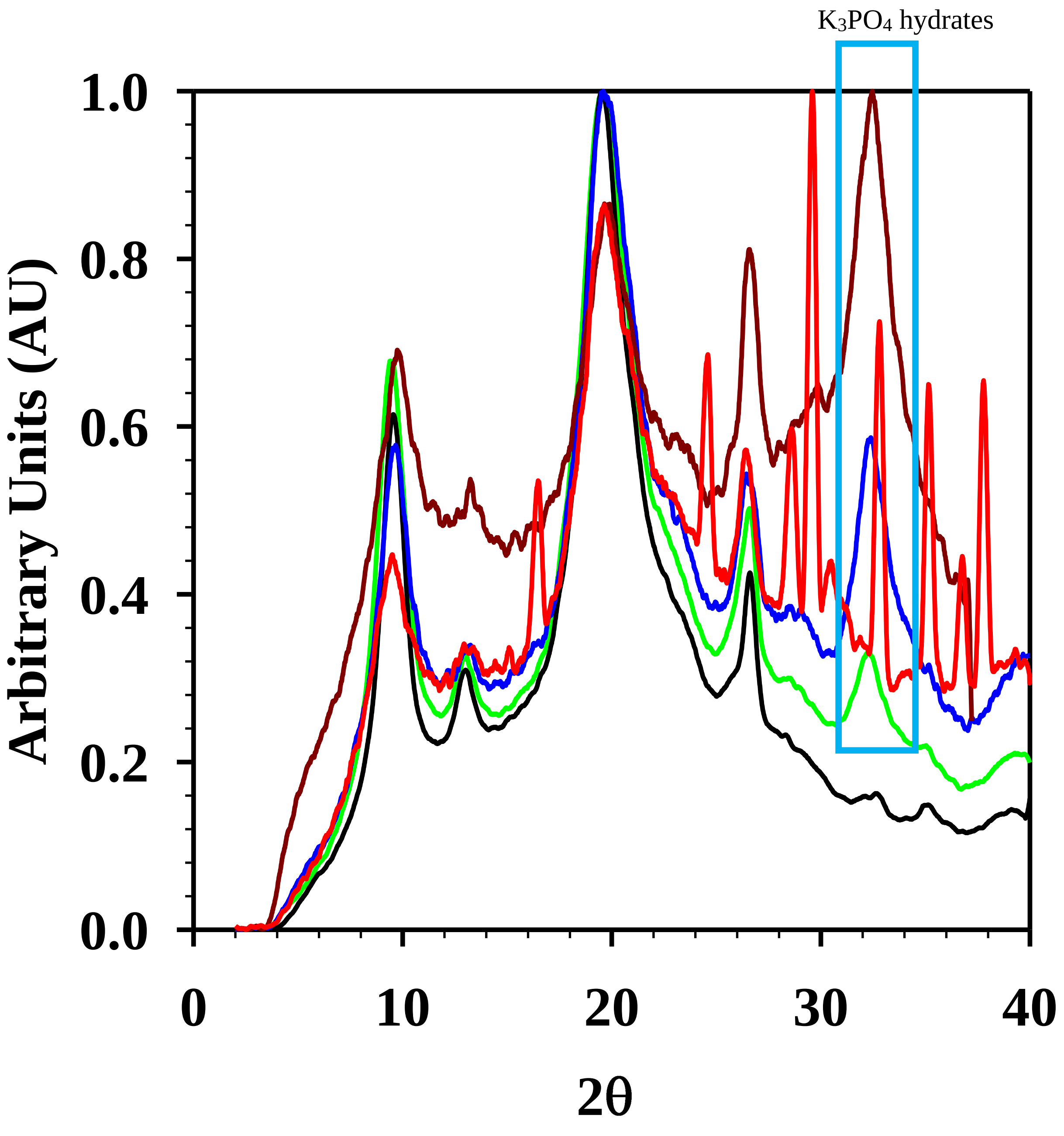


**Figure S2.** pXRD of chitin materials obtained utilizing $K_3PO_{4\,aq}$ for deproteinization. Red: shrimp shell biomass, Black: IL-chitin; Lime Green: IL-chitin$_{dry/K3PO4}$; Blue: IL-chitin$_{swollen/K3PO4}$; Burgundy: chitin obtained in accordance with reference [28].

**pXRD from Polysorbate 80**

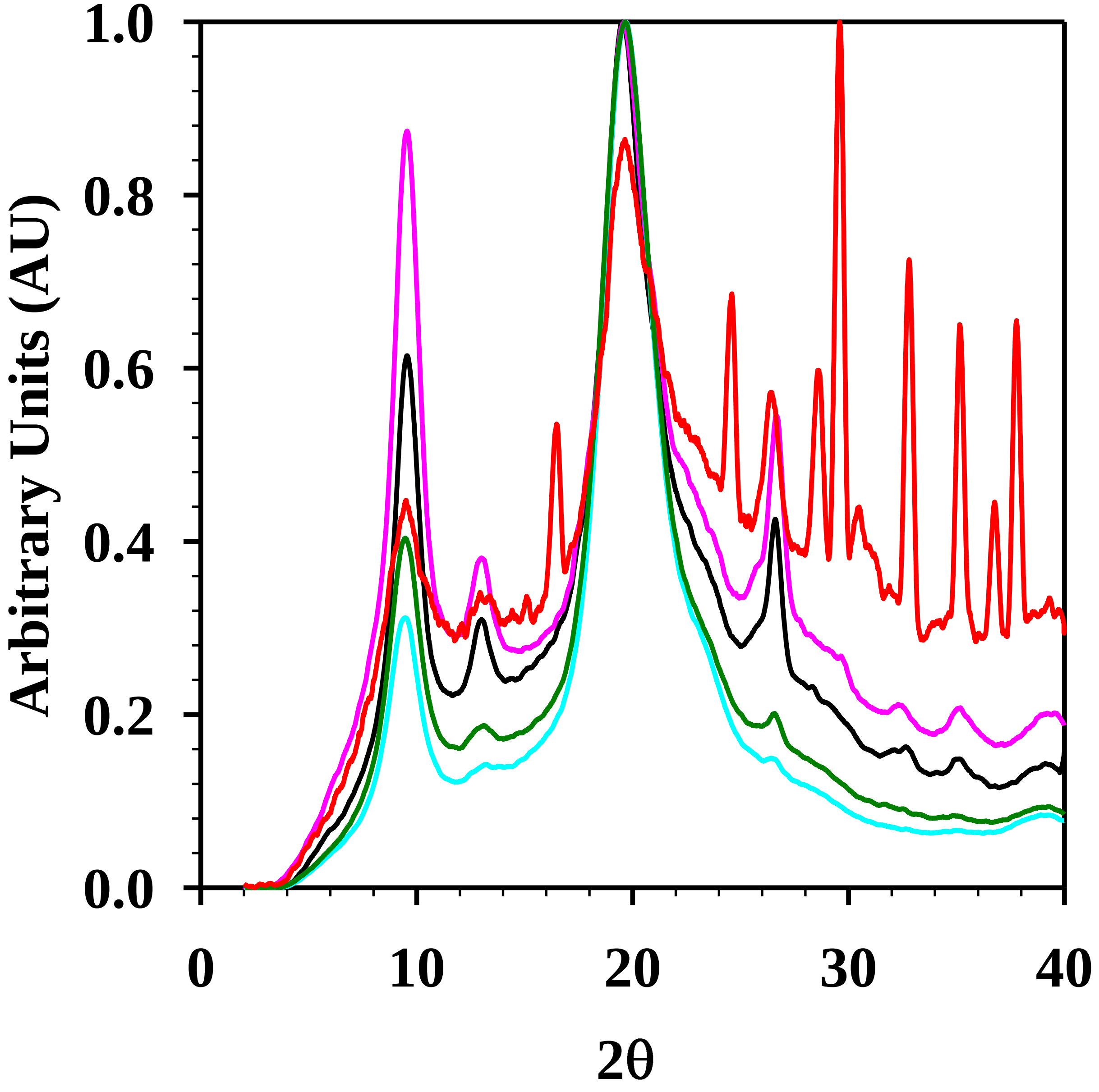


**Figure S3.** pXRD from chitin utilizing Polysorbate 80 for deproteinization. Red: shrimp shell biomass; Black: IL-chitin; Dark Green: IL-chitin$_{dry/Polysorbate\ 80}$; Pink: IL-chitin$_{swollen/Polysorbate\ 80}$; Aqua: chitin obtained by coagulation of IL-chitin solution into Polysorbate 80 instead of water.

**List of Proteins in Chitin before Deproteinization**

The mass spectrometry data were processed using Protein Lynx Global Server (PLGS) version 2.5.3 (Waters, Milford, MA) and then analyzed. Analysis was done first using IdentityE (Waters Corporation, Milford, MA; version iaDBs: 2.112.0.0), and then Scaffold software. IdentityE was set up to search null assuming the digestion enzyme trypsin. IdentityE was searched with a fragment ion mass tolerance of 0.025 Da and a parent ion tolerance of 0.0100 Da. Dehydration+ST of, amidation+C-TERM of, deamidated of asparagine and glutamine, oxidation of methionine, carbamidomethyl of cysteine and phosphoryl STY of serine, threonine and tyrosine were specified in IdentityE as variable modifications.

Scaffold validated MS/MS based protein identifications by analyzing tandem mass spectrometry data that has been processed by the search engine and transformed the search engine scores into statistical probabilities that makes protein identifications easier to validate. Peptide identifications were accepted if they could be established at greater than 20.0% probability by the Scaffold Local FDR algorithm. Protein identifications were accepted if they could be established at greater than 20.0% probability and contained at least 1 identified peptide. Protein probabilities were assigned by the Protein Prophet algorithm by the software. Proteins that contained similar peptides and could not be differentiated based on MS/MS analysis alone were grouped to satisfy the principles of parsimony. Proteins sharing significant peptide evidence were grouped into clusters.

*Pvannamei database*: 152 proteins in 108 clusters. Cluster of Fast-type skeletal muscle actin, Cluster of Myosin heavy chain type 1, Cluster of Hemocyanin, Cluster of Sodium/potassium-transporting ATPase subunit alpha, Cluster of Leucine-rich repeat flightless-I-interacting protein 2D, Cluster of Myosin heavy chain type 2, Cluster of Alpha-2-macroglobulin, Cluster of Beta-catenin, Cluster of Prophenoloxidase, Cluster of IKKepsilon1, Beta-1,3-glucan-binding protein, Cluster of Janus kinase, Cluster of Pyruvate kinase, Clathrin heavy chain, Retinoblastoma family-like protein, Argonaute 1, Cluster of Heat shock cognate 70, Toll protein, Vascular endothelial growth factor receptor, Argonaute, Ryanodine receptor, Heat shock 70 kDa protein cognate, IL-16-like protein, Flotillin-2, Arsenite-resistance protein 2-like protein, AMP-activated protein kinase subunit gamma, Dicer 2, Domeless, Polephole-like protein, tropomyosin, Nitric oxide synthase, Phosphatidylinositol 3-kinase, Phosphoenolpyruvate carboxykinase, Sterile-alpha and armadillo motif containing protein, Heat shock protein 60, Argonaute 2, Timeless protein, L-lactate dehydrogenase, Mitogen-activated protein kinase 4, C-terminal-binding protein, Cytochrome P450,

Glyceraldehyde-3-phosphate dehydrogenase, Calcium-transporting ATPase, Serpin8, Serpin 3, Toll2, Apoptosis signal-regulating kinase 1, Importin subunit alpha, F0-ATP synthase b-chain, Adenosine monophosphate deaminase 2 (Fragment), ATP synthase subunit beta, TNFSF. Random Sequences 43, 50, 68, 88, 127, 182, 183, 191, 213, 240, 278, 307, 348, 366, 370, 373, 391, 434, 472, 481, 491, 500, 511, 543, 553, 560, 571, 582, 588, 605, 623, 647, 654, 660, 666, 673, 715, 722, 731, 738, 750, 753, 762, 766, 804, 807, 826, 827, 860, 865.
